# A Comprehensive Students-as-Teachers Program: Developing and Empowering Future Medical Educators

**DOI:** 10.1007/s40670-024-02062-4

**Published:** 2024-06-18

**Authors:** Selina Noramly, Linda Waggoner-Fountain, Meg Keeley, Deborah Barry

**Affiliations:** University of Virginia School of Medicine, Charlottesville, VA USA

**Keywords:** Students as teachers, Teaching assistants, Course development

## Abstract

We describe the development of two formats of a Students as Teachers (SaT) program that was designed to train fourth-year medical students as near-peer teachers in the pre-clinical classroom. This program has served 191 students since its inception in 2017 through a 2-week credit bearing elective or an evening workshop series. We describe key elements of the courses and positive outcomes of our program within a learning communities’ framework. We present these data for other institutions interested in creating their own SaT program.

## Background

Teaching medical students how to teach is a rapidly expanding area of medical education. At most institutions, the primary function of these efforts is to improve the teaching of students who serve as near-peer teachers or tutors. Several recent scoping reviews of medical students-as-teachers (SaT) programs [1–4] outline the multiple reasons for their growth. Medical student teaching programs can positively impact the student learners, the medical school program, and the medical student teachers themselves. Medical student teaching assistants (TAs) can provide near-peer teaching as well as coaching to other students. Many medical educators experience challenges with teaching difficult concepts despite being experts in their field [5] while TAs may be more accessible to certain groups of student learners as they focus on the essentials of what needs to be learned at that moment. In this way, medical student teaching programs can impact organizational effectiveness. TAs enhance their own teaching efficacy and typically consolidate their knowledge of the curricular content [4]. Serving as a TA can help lay the foundation for professional identity formation as a medical educator. Experience as a TA may encourage a medical student to pursue one of the increasing number of residencies and fellowships that have educational tracks [6–8].

Teaching assistantships have existed in the basic and applied sciences for over 50 years as a way for PhD students to both fund their education as well as teach service courses for their departments [9]. However, in medicine and as well as in other disciplines, SaT Programs, lack systematic programing to train the TAs in pedagogical content knowledge. Most TAs participate in some sort of program- which can vary from a one-day workshop, learning communities that meet weekly to discuss teaching assignments, teaching mentors, participation in teaching seminar, observations of award-winning faculty, or year-long courses aimed to enhance teaching skills [10, 11]. However, our medical students did not have access to any department or university wide programing to prepare them to serve as TAs. This led to the development of our own University of Virginia School of Medicine teaching courses as described below.

## Activity

We designed a program to prepare medical students as future patient and peer educators through a comprehensive and robust training program to assist students in developing their teaching philosophy, practice skills-based teaching, and complete a final medical education teaching and/or curriculum-based project [12, 13]. Our students apply through a competitive application process for a TA position in the pre-clerkship courses during their final phase of the curriculum. They can also contribute to the pre-clerkship and clerkship curriculum through supervised projects and/or apply to be a TA in the anatomy laboratory. Students may receive up to 8 weeks of elective credit depending on their time commitment. This paper describes the evolution of the program into the current course formats as well as the positive outcomes of student participation.

## Course Formats

This program was developed utilizing a backwards course design during a week-long intensive Course Design Institute (https://cte.virginia.edu/programs-grants/course-design-institute), and first piloted in 2017 [14]. Since then, three formats of the course have been offered, a two-week intensive in-person course with 40-50 contact hours, a two-three evening workshop series meeting for 6-10 contact hours, and an online two-week intensive delivered during the COVID-19 pandemic.

**Fig. 1:**
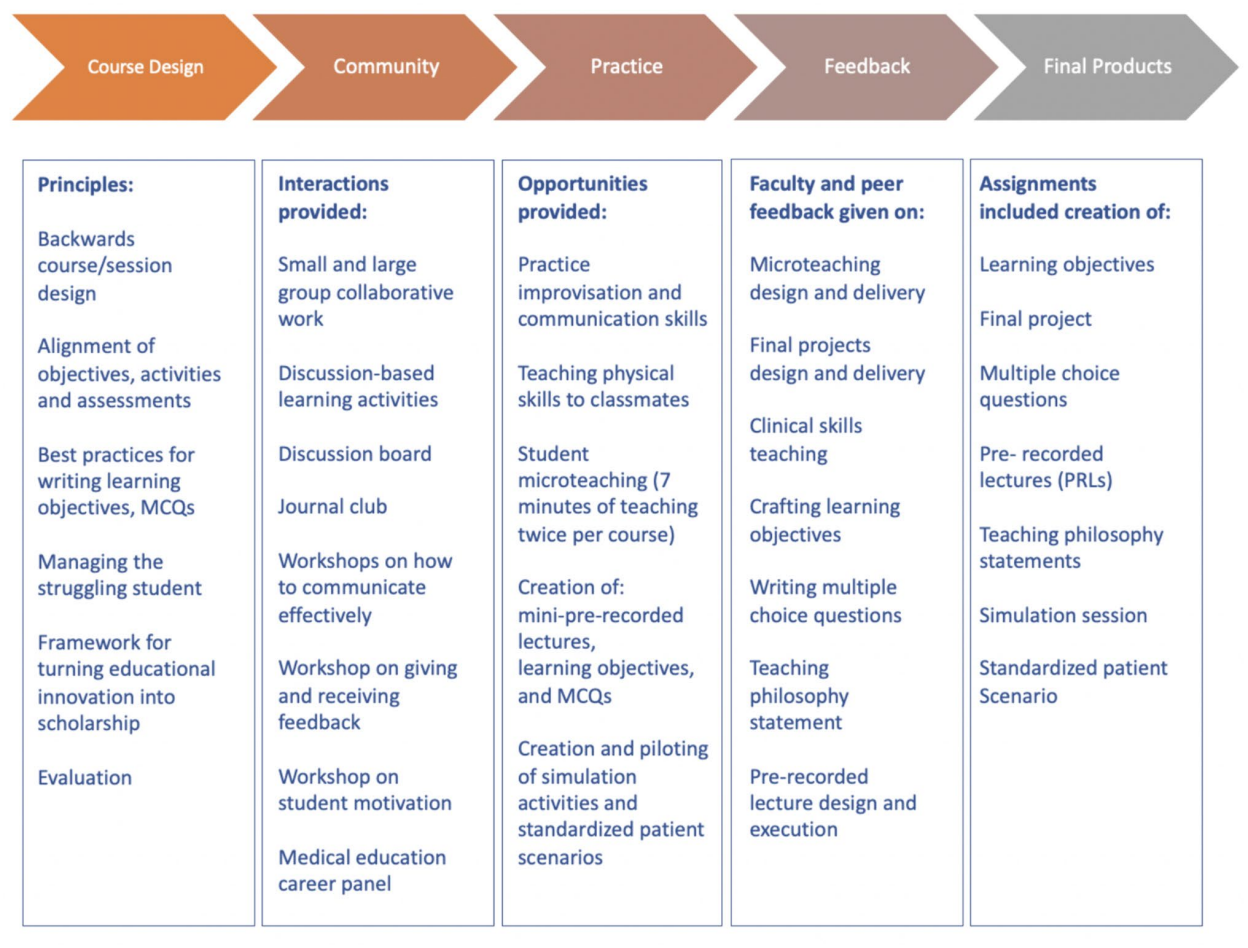
A learning communities framework was chosen to guide our course development and implementation as our medical school utilizes this organizational schema throughout our four-year curriculum to support student learning

Each course format includes the opportunity for students to develop their teaching philosophy, practice skills-based teaching, participate in microteaching small group sessions with peer and faculty feedback, write and review multiple choice questions, and create and produce pre-recorded lectures (Fig. 1). Students within the intensive format courses develop their communication skills through a series of improvisation workshops; design, pilot and test simulated patient encounters; and create a teaching related session/product while receiving feedback from peers and faculty. This course also highlights the use of educational technology in the medical school classroom, provides practice opportunities for skill development, and reinforces the use of technology to support the development of pedagogical content knowledge.

## Results

In total, 191 students have completed the training, with 136 students serving as TAs after completing the program. Approximately half of the participants took the two-week intensive vs. the two half-day workshops. The workshop series have become increasingly popular as students have many options when setting their elective schedules. Participants have created high quality pre-clerkship classroom and course materials in all pre-clerkship courses, including pre-recorded lectures, over 300 multiple-choice questions, and document or PowerPoint handouts. Students have led or co-taught with faculty more than 25 active learning sessions within the pre-clerkship courses and hosted more than 100 optional post-class review sessions which receive high praise from students. Clerkship, study groups/materials, and wellness resources have also been created and disseminated. Faculty have reported on the high-quality of student created materials, as well as increased engagement of teaching assistants after program participation. Students routinely cite TAs as a valued course resource in course evaluations. A sample of student feedback, 2 courses over 3 years related to TAs were evaluated, with 288 comments in praise of TAs (Table 1). There is positive synergy of this program with our student-led and staffed peer mentoring program in that participants often volunteer as peer mentors who work with self-identified students on any perceived academic challenges. Ninety-three percent of teaching award recipients were graduates of the training since its inception.

**Table 1:** A review of student evaluations of courses yielded 5 themes in praise of teaching assistants.

**Theme**	**Example Quote**
**Availability**	“Involvement and availability of the TAs this system was amazing. I appreciated the specialized emails and review sessions the TAs held throughout the system.”
**Materials**	“The ECG handout was amazing – I think system leaders should look into distributing that during the ECG sessions”
**Teaching Skills**	“They are some of the best TAs I have ever seen. They should be proud of their work done and their understanding of the various types of students. Honestly, they are better than most instructors and I strongly believe that they should be the model other practicing physicians aspire to when teaching medical students. The highest of praise to them and whoever selected them. They honestly put so much work into it and I have nothing to say but thank you”
**Review Sessions**	“I thought the TAs did a great job during the reviews to put all the information we acquired throughout the course into part of a big picture”
**Critical for student learning**	“I don’t think I would’ve passed this system without the TAs. Their weekly review sessions were high yield and their exam review sessions with board-style questions really challenged us to make connections between the different disease processes and medications we were learning. Also, their energy was great. I hope to be as well-versed and confident as they are!”

## Discussion

The development and implementation of the SaT program following a systematic model to improve the pedagogical content knowledge of our medical students has had a significant impact on student satisfaction within our pre-clerkship curriculum, as well as faculty satisfaction with the quality and involvement of the teaching assistants [15] (Table 1). Students often cite the teaching assistants as “essential to their learning” and a “strength of the course”. As this program has grown over the years, it has become a required part of medical student training prior to completing a teaching assistantship. Barry and Noramly (2020) analyzed the reflective teaching statements and classroom teaching of a sample of TAs, finding that programs graduates can actualize their teaching beliefs within the classroom [16]. While the long-term outcomes of the SaT program are not fully known, 80% of program participants cite their desire to pursue a career in academic medicine. Early graduates of one SaT program have been more involved in medical education in their careers 6-9 years after graduation than students who did not take the SaT, including students who had expressed interest in participating in the SaT but did not do so [17]. In PhD programs, participating in TA programs in associated with an increased likelihood of obtaining a teaching focused career, indicating the importance of this training in career choice and professional identity formation [18]. Future research should investigate the career development of medical students who participate in these or other pedagogical training programs, further investigating the impact of participation on professional identity formation.

Over 60 students have participated in the elective or workshop series because of their interest in developing their teaching skills in advance of starting their residency programs but were not registered as part of pre-requisite training for a TA position. They cite their passion for teaching, interest in medical education and the importance of teaching assistants for their own learning in medical school. Increased offering of the elective to include those students completing medical education curriculum projects and anatomy laboratory-based electives has allowed for the development of high- quality classroom deliverables in addition to several projects that have resulted in conference presentations.

The Learning Communities Framework, outlined in Fig. 1, highlights the alignment of specific course elements to each aspect of the model. While our program focused on specific elements of developing pedagogical content knowledge within a learning community, the program could be adapted and delivered by other medical schools to fit within their curricular frameworks. Future iterations of the program will include the scholarship of teaching and learning. We aim to encourage our students to engage with research related to teaching and learning as well as disseminate their innovations.

## Conclusions

The varied delivery models of our SaT program allows us to reach a large number of interested students, prepares future medical educators and results in both high quality educational experiences and deliverables. Future research regarding SaT programs should investigate long-term outcomes of program graduates in their residency placements, while encouraging wide participation within medical schools to best prepare our future medical educators for success within residency and beyond.

## Data Availability

The data that support the findings of this study are available on request from the corresponding author, DSB. The data are not publicly available due to their containing information that could compromise the privacy of research participants.
